# Radio-frequency current drive for thermonuclear fusion reactors

**DOI:** 10.1038/s41598-018-27996-9

**Published:** 2018-07-09

**Authors:** A. Cardinali, C. Castaldo, R. Cesario, L. Amicucci, A. Galli, F. Napoli, L. Panaccione, C. Riccardi, F. Santini, G. Schettini, A. A. Tuccillo

**Affiliations:** 1 ENEA, Fusion and Nuclear Safety Department, C. R. Frascati, Via E. Fermi 45, 00044 Frascati (Rome), Italy; 2 Università Roma La Sapienza, Dipartimento di Ingegneria, Rome, Italy; 3 Università Milano-Bicocca, Dipartimento di Fisica, Milan, Italy; 4 Università Roma Tre, Dipartimento di Ingegneria, Rome, Italy

## Abstract

Principal research on energy from thermonuclear fusion uses Deuterium-Tritium plasmas magnetically trapped in toroidal devices. As major scientific problem for an economic (i.e., really feasible) reactor, we must understand how to lead strongly heated plasmas to sustain a high fusion gain while large fraction of current is self-produced via the presence of strong pressure gradient. To suppress turbulent eddies that impair thermal insulation and pressure tight of the plasma, current drive (CD) is necessary. However, tools envisaged so far in ITER (International Thermonuclear Experiment Rector) are unable accomplishing this task that requires efficiently and flexibly matching the natural current profiles of plasma. Consequently, viability of a thermonuclear reactor should be problematic. Multi-megawatt radio-frequency (RF) power coupled to plasma would produce the necessary CD, but modelling results based on previous understanding found difficult the extrapolation of this CD concept to reactor conditions of high temperature plasma, and greater flexibility of method would also be required. Here we present new model results based on standard quasilinear (QL) theory that allow establish conditions to drive efficiently and flexibly the RF-driven current at large radii of the plasma column, as necessary for the goal of a reactor.

## Introduction

**Figure 1:**
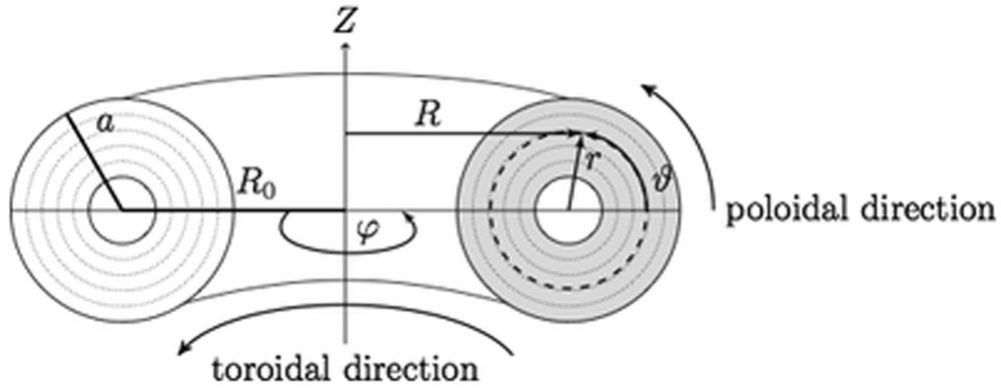
Scheme of axisymmetric toroidal column of plasma, magnetically trapped in a tokamak machine by static magnetic field, **B**_T_, produced by a solenoid surrounding the column. *R* and *r* are, respectively, the major and minor radial coordinates, *R*_0_ indicates the position of the column axis, and *a* is the last closed magnetic surface (LCMS).

Research on thermonuclear fusion has to face formidable scientific challenges for managing to achieve, directly on Earth, *true* nuclear energy via the major role that, with respect to fission, the potential of the fundamental strong nuclear force plays in fusion reactions. The principal obstacle derives from not being fusion reactions a spontaneous mechanism allowed by nature on the planet, which is instead the case of all energetic options available so far. New energy would allow using, for safety of biosphere, the renewable and unlimited resource of Deuterium, abundant in the planet’s waters. Main research on fusion energy utilises toroidal plasmas magnetically trapped in toroidal machines^1^ (*tokamaks*), see Fig. 1.

The plasma forms a ring that is the secondary winding of a transformer that needs for initiating by induction a current that in a reactor, with the aid of heating and current drive (CD) systems, would reach high intensities (>10MA) necessary for hugely heating the plasma (up to about 100 millions of degrees) in order to establish thermonuclear fusion of Deuterium-Tritium plasma by winning the electric repulsions of the ions. (Tritium is self-produced by using suitable material for the chamber of machine).

The over all magnetic field vector, **B**, necesary for confining the plasma results from the **B**_T_ field and the perpendicular (poloidal) component **B**_⊥_ generated by the plasma current. Consequently, in the lack of the Lorentz force component, plasma particles are free of moving along the magnetic lines. Owing to contribution of the poloidal component, the magnetic lines form helical paths around the torus that further enhance the plasma insulation. The number of poloidal turns of a magnetic line along a full toroidal turn defines the safety factor (in the cylindrical limit): $q=\frac{r{B}_{T}(r)}{{R}_{0}{B}_{\perp }(r)}\approx r\frac{{B}_{T}}{{R}_{0}{B}_{\perp }}$, and the shear (*s*) of the magnetic lines has the expression: $s=r\frac{dq}{dr}.$ Defining the normalized minor radius of the plasma column as: $x\equiv \frac{r}{a}$, the heating power naturally produces a warm, dense and thermally better-insulated plasma core (at *x* ∼ 0), and a relatively colder and more rarefied plasma edge (at *x* ∼ 1). Low magnetic shear (*s* ∼ 0) is condition useful for producing turbulence suppression and, in turn, thermal insulation improvement of the heat and particle content of the plasma colum^1,2^.

There is general consensus that, for being feasible, a reactor must be economic, i.e., the huge costs of the heating and CD systems should be mandatorily reduced by exploiting a strong fraction of current that the plasma self-produces steadily via particle transport effect (*bootstrap*). The latter occurs in the presence of a pressure gradient across the plasma column, for given value of the confinement magnetic field. The stronger pressure gradient occurs mostly at large radii, namely at around the position of the so-called *pedestal*, where the pressure tends to naturally and abruptly vanish^1,3^. The pedestal builds-up in the H (high)-confinement mode that spontaneously develops under strong enough values of the injected heating and CD powers^4^. This regime is generally considered useful for the goal of a reactor.

A major problem consists in the fact that the *bootstrap* current density radial profile, *j*_BS_(*r*), cannot be set independently of the pressure profile because is determined by the confinement performance; the latter, in turn, is affected by the current density profile. Therefore the primary need of a reactor of exploiting a strong bootstrap current fraction relies upon the active control of the current density profile^1,2,5–7^.

Important experiments of JET (Joint European Torus)^8^ supports the need of actively tailoring the current profile including the pedestal radial region^6^. Utilising strong neutral beam (NB) power (20 MW), these experiments revealed the attractive ability of plasma self-organize conditions useful for the goal of a reactor^6,7^. In fact, a high *j*_BS_ fraction built-up near the radial plasma periphery did allow lowering the shear and reduce turbulence^6^. Consequently the plasma was able to build self-sustaining a phase of good thermal insulation. This phase collapsed after a few seconds, owing to the continuing diffusion towards the centre of the current fraction induced by the transformer. This insufficiency of the *j*_BS_ contribution during the best phase of confinement prevented self-maintaining appropriately low the local shear against the onset of detrimental turbulence, whose onset caused indeed collapse of the *j*_BS_ fraction and, consequently, of confinement. This problem would have been prevented by means of a CD contribution actively produced at large radii, possibly corresponding to a relatively small fraction of *j*_BS_^6^. The availability of an active CD method with coverage at large radii would have facilitated the progress of the confinement performance experiments of JET.

Tools envisaged so far for ITER (International Thermonuclear Experiment Reactor), now in construction phase^4^ are unable to efficiently cover the outer radial half of plasma. They are indeed based on NB and electron-cyclotron radio-frequency (RF) powers that have poor efficiency and flexibility mainly approaching the pedestal radial layer. This deficiency can be solved by the lower hybrid current drive (LHCD)^9,10^, because major problems encountered with this concept for long time in tokamak experiments have been now solved, as discussed more ahead.

The relevant CD method utilises lower hybrid (LH) waves, i.e., quasi-electrostatic plasma modes that in tokamak plasma have frequencies of several gigahertz and have the special feature of efficiently transferring momentum to plasma electrons that are free of moving along the toroidal direction. An antenna useful for performing this task consists of a phased array of rectangular waveguides, fitting the relatively narrow gaps of the toroidal magnet for natural implementation in a reactor.

LH waves are damped via Landau wave-particle resonance (in the phase and drifting velocities, respectively) with a tail of the electron distribution function (EDF)^9,11^:

1 $${u}_{{\rm{\Phi }}//}\equiv \frac{{\omega }_{0}}{{k}_{//}}=\frac{c}{{n}_{//}}\approx 3{u}_{the}$$

where: ${u}_{{\rm{\Phi }}//}$ and *k*_//_ are, respectively, the components of wave phase velocity and wavevector parallel to **B**_0_, *c* is light speed, ${n}_{//}\equiv \frac{c{k}_{//}}{{\omega }_{0}}$ is the parallel refractive index component, and ${u}_{the}\equiv \sqrt{\frac{2{k}_{B}{T}_{e}}{{m}_{e}}}$ is the electron thermal velocity, where *k*_B_ is the Boltzmann constant, *T*_e_ is the electron temperature, and *m*_e_ is the electron mass.

**Figure 2:**
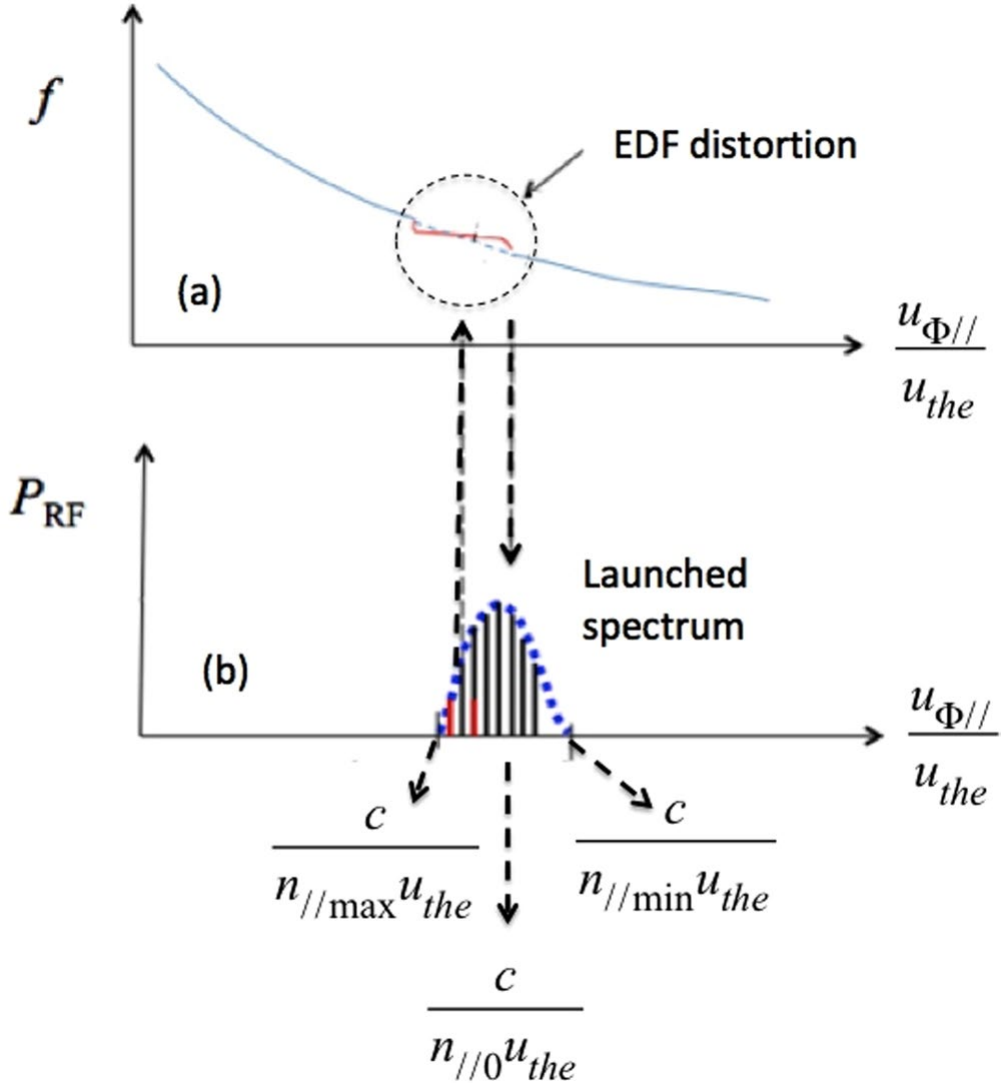
(**a**) Electron distribution function (EDF). (**b**) Gaussian antenna spectrum of multi-megawatt radio-frequency power (in arbitrary units), coupled to plasma at several gigahertz, useful for producing the LHCD effect^9^.

Figure 2 schematizes the LHCD concept^9^, proven by experiment on PLT^10^ (Princeton Large Torus), and successfully extrapolated on FTU (Frascati Tokamak Upgrade) to reactor-graded, high plasma densities^12–15^.

Accordingly to Eq. 1, considering a Gaussian spectrum with *n*_//0_ and Δ*n*_//_ parameters (Δ*n*_//_ is kept at half of the peak value), plasma electrons Landau-resonate more intensely with spectral components with slower phase velocity. LH waves are electron plasma modes particularly prone to drive current, i.e, to transfer energy and momentum to plasma electrons along the direction of the confinement magnetic field, where they are free to drift. Consequent to slow-wave behaviour of LH modes (which have indeed refractive index component: *n*_//_ > 1), electrons flowing with speed close to the phase velocity of the wave *see* its electric field as almost constant. The particles can thus be accelerated by the wave with the result, via collisions, of an irreversible transfer of energy and momentum to the electrons, constituting the LHCD effect.

Previous works found that too high electron temperatures envisaged for DEMO at the pedestal radial layer would prevent the RF power coupled by the antenna to usefully reach the pedestal region itself and penetrate in the plasma bulk^16,17^. We here present new results indicating that, by acting on an antenna parameter not adequately considered so far, namely the *n*_//_, spectral width, Δ*n*_//_ the LH power deposition should usefully occur radially deeper in reactor plasmas, under sufficiently high values of the toroidal magnetic field.

The need of a current profile control system capable of covering the outer radial outer half of plasma including the pedestal radial layer, crucial for stability, should be satisfied by the LHCD option. Indeed, obstacles met for decades represented by too high densities of reactor plasmas have been more recently removed^12^, while here it is indicated how to solve the remaining major problem represented by too high electron temperatures. Therefore the LHCD method should be reconsidered as a priority for ITER and DEMO.

In order to help understanding the relevant mechanism considered here, Fig. 2b shows the *n*_//_ power spectrum of LH waves launched by the antenna, centred at *n*_//0_, with spectral components in the interval (*n*_//max_ − *n*_//min_). Spreading radially towards the warmer regions of the plasma, the slowest components of the spectrum (with *u*_ϕ//_ ∼ *u*_ϕ//min_ i.e., *n*_//_ ∼ *n*_//max_) are beginning to first to interact more intensely with the plasma, in accordance with Eq. 1. Consequent to accelerated population, some EDF flattening occurs as shown in Fig. 2a. This distortion determines the modality of the wave packet absorption^9^ and evidences the key role of the spectral width discussed here for thermonuaclear plasma regimes.

In view of the ease in grasping the concept of LHCD, to obtain my modelling the shape of the driven current density profile, *j*_LH_, is a difficult task. This can be accomplished by proper numerical approach able to display aspects of LHCD mechanism remained hidden so far.

The new method presented here for enabling LHCD would make less challenging the goal of a reactor. Indeed previous believing considered that the necessary conditions of a high fusion gain and stability in a ractor would be produced by external heating and CD power systems, but their huge costs would prevent the construction of demonstration experiments. Economical constraint of a reactor encourages the efforts for promoting and optimising the selforganisation features of tokamak plasmas for approaching burning plasma conditions. The solution of the latter major problem would be facilitated by exploiting the LHCD method in the new light presented here.

## Results

The concept of current drive based on the acceleration of plasma electrons by externally launched RF power coupled to LH waves requires to calculate the balance between competing effects, from one side, by the LH wave–particle interactions (Eq. 1) and related diffusion in the velocity space; from the other side, by collisions that tend restoring the Maxwellian shape of EDF. These effects are described by the quasi-linear (QL) theory^18–21^ and the EDF evolution is described by the Fokker-Planck equation^9^:

2 $$\frac{\partial f}{\partial t}=C(f,f)+C(f,{f}_{i})-\frac{\partial f}{\partial {\bf{v}}}{S}_{w}$$

where *C*(*f*, *f*) represents the self-collisions of electrons, *C*(*f*, *f*_*i*_) the electron scattering of ion distribution *f*_i_, and *S*_w_ is the wave-induced flux that depends on both the nature of the wave-particle interaction and the velocity-space gradient of the EDF, and is expressed by:

3 $${S}_{w}\equiv -\,{D}_{QL}\frac{\partial f}{\partial {\bf{v}}}$$

where *D*_QL_ is the quasilinear diffusion coefficient.

In slab plasma geometry where density and temperature gradients are directed along the *x*-direction defined above, in steady-state conditions the spectral components *P*(*n*_//_) of the LH wave power are absorbed accordingly with the equations^22^:

4 $$\frac{dP}{dx}={{\rm{\Gamma }}}_{QL}P$$

where Γ_QL_ is the quasilinear damping rate inferred from the 1-D distribution function of plasma electrons.

Standard analytical calculations show that the damping rate should be reduced by means of a larger RF power spectral density that can be produced by coupling: i) a stronger RF power for given launched *n*_//_ spectrum of antenna, as shown in ref.^23^, ii) a narrower *n*_//_ spectral width for given RF power, which is considered here. To reduce the wave damping is necessary for removing the obstacle to wave propagation represented by too high temperatures possibly occurring even at large radii of the reactor plasma column, as found in ref.^16,17^. In this regard the option of using strong RF power for given launched *n*_//_ spectrum of antenna is not viable because in contrast with safe antenna operations^24^. Consequently this work focuses on the use of a suitable *n*_//_ spectral width of antenna.

In order to attack the problem, analytical 1-D approach generally produces too crude approximations incapable of taking into account important details of the quasilinear diffusion coefficient. Therefore, to assess the LHCD profiles in practical cases, as shown hereafter, numerical approach has been used based on 3-D ray-tracing modelling for taking into account the effects of wave propagation in toroidal geometry, and retaining effects of RF diffusion in 2-D in *u*_//_ and *u*_⊥_ of electron velocity. Short mention of some results contained in the paper is given in ref.^25^ which however does not consider the role of the toroidal magnetic field for preventing the parasitic broadening of the launched spectrum. This information and detailed exposition necessary for better understanding the results have been given here.

**Figure 3:**
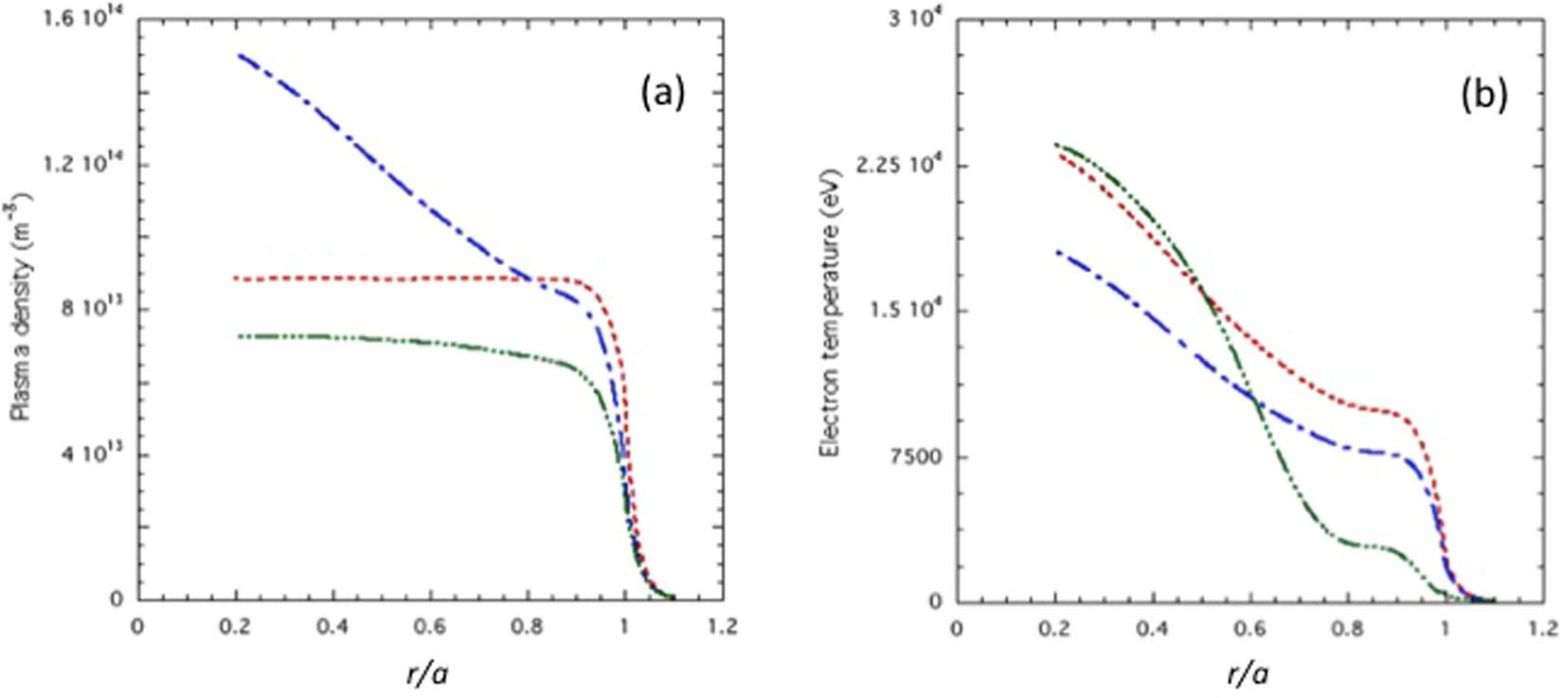
(**a**) Electron plasma density (*n*_e_) radial profiles envisaged, respectively, for the DEMO *pulsed* regime (blue curve), and the *steady-state* regimes of DEMO (red curve) and ITER (green curve). (**b**) Electron temperature (*T*_e_) profiles of the *pulsed* (blue curve) and *steady-state* (red curve) regimes of DEMO, and the *steady-state* regime of ITER (green curve).

Examples of radial plasma profiles of the electron plasma density and temperature envisaged for the DEMO reactor^16,26^ and utilised for numerical modelling considered here are shown in Fig. 3.

DEMO aims at demonstrating the technological and economic feasibility of the new energy by the first working fusion power station. The plasma regimes, respectively, *pulsed* (blue curves) and *steady-state* (red curves) are envisaged, both exhibiting a high H-mode of confinement^16,26^. The pulsed regime has the plasma current density profile not fully relaxed, its duration is of about a couple of hours and has as scope to save the costs of the heating and CD power systems^3^. Steady-state regime would instead operate with continuity as ideally required by a reactor [T.C. Luce, Realizing steady-state tokamak operation for fusion energy, Phys. of Plasmas 18, 030501 2011].

Works mentioned in the Introduction found an LH power deposition too far out in the DEMO plasma^16,17^, considering as input electron temperature profiles like those shown in Fig. 3. Namely, too high values at around the pedestal radial layers (*T*_e_ = *T*_e_0.9_ ∼ 8 keV at normalised minor radius: *x* ∼ 0.9) would cause full deposition at the very edge of the coupled RF power^16,17^. Conversely we show hereafter that considering DEMO profiles of Fig. 3, this problem does not occur thanks to exploitation of a more suitable antenna spectrum.

The dependence of the toroidal magnetic field on *R*, in the equatorial plane, see Fig. 1, is: *B*_T_  = *B*_T0_/*R*. Consequently, the plasma has regions with highest field side (with *B*_T_ = *B*_THFS_ ≈ 8.5 T) and lowest field side (with *B*_T_ = *B*_TLFS_ ≈ 5.5 T) situated, respectively, at the plasma edge layers: *R*_0_ *−* *a* and *R*_0_ + *a*. Further relevant parameters are summarized in the Method Section. Steady-state regime profiles of ITER (green curves), exhibiting lower values of plasma density and temperature, are also displayed for comparison in Fig. 3.

### Antenna spectra

**Figure 4:**
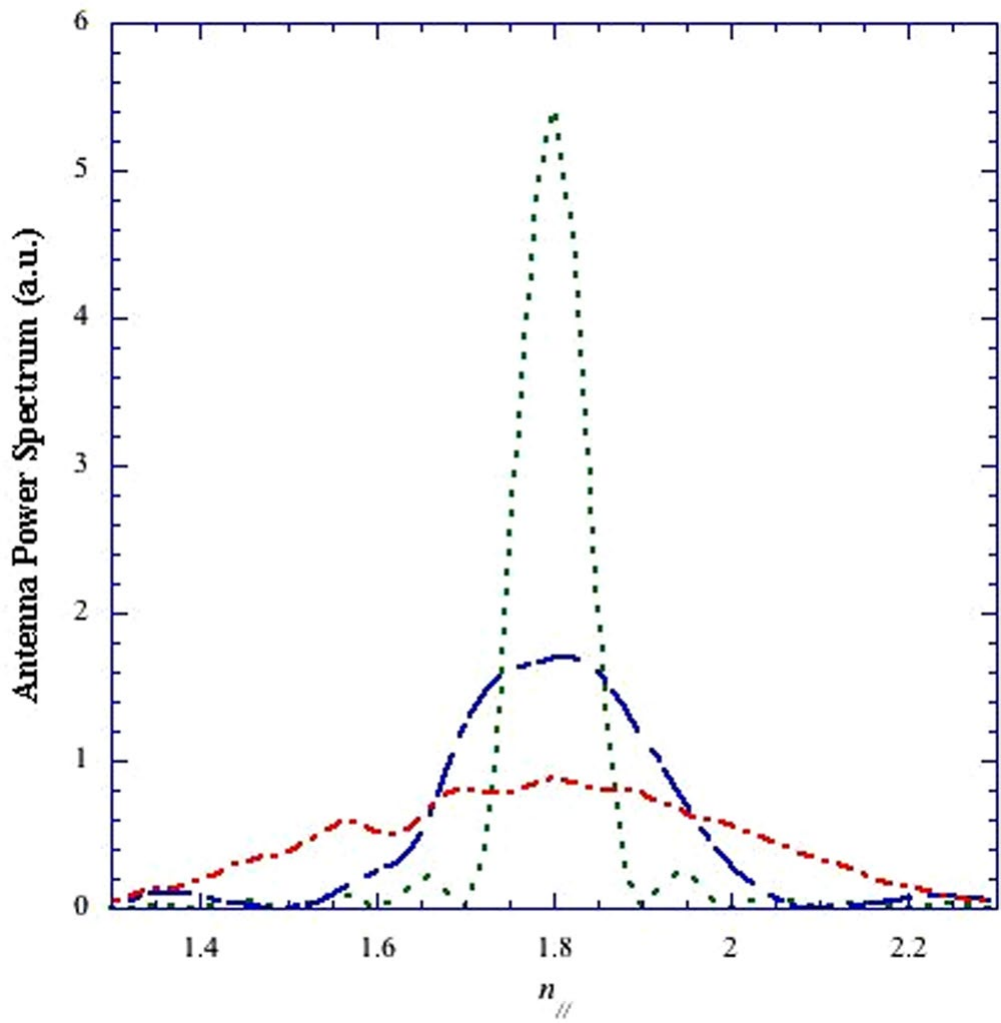
Antenna spectra numerically calculated^26^ considering modules of LHCD antenna proposed for ITER^4,28^, see the Method Section. Suitable phasing and feeding of the waveguides produce spectra centred at same *n*_//0_ (=1.8) but with three different widths, respectively, Δ*n*_//_ = 0.085 (green curve), Δ*n*_//_ = 0.33 (blue curve) and Δ*n*_//_ = 0.58 (red curve).

The antenna spectra considered for modelling the j_LH_ profiles are displayed in Fig. 4. The peak value (*n*_//0_ = 1.8) has been chosen in order to satisfy, for the assumed kinetic profiles of Fig. 3, the LH wave accessibility condition^11^. These spectra have been obtained using the numerical tool GRILL-3D^27^ able to treat however complicated antenna geometries of phased arrays of waveguides. The same antenna modules shown in the design proposed for a LHCD system for ITER^28^ have been considered, as detailed in the Method section that also describes the assumed antenna geometry that faces the DEMO plasma.

### Compatibility with QL theory limit

In order to satisfy compatibility with QL theory, the local RF power density should be sufficiently low. This condition has been verified accordingly with criterion described in ref.^18^ that allows retaining the approximation of linearised particle orbit limit for all cases obtained considering as input the RF power spectra shown in Fig. 4 (referring to a same RF power coupled by the antenna). It has been thus sufficient to check QL theory validity for the case with maximum RF power density, i.e., the one having minimum spectral width (Δ*n*_//_ = 0.085).

For an evolving spectrum of dispersive waves, this pattern will persist for a limited lifetime (*t*_L_) to be compared to the bounce time (*t*_B_) of a particle in this pattern. The latter is the time spent by a particle to reverse direction and come close to the initial position. QL limit requires that: *t*_B_ >> *t*_L_. Consequently, the field pattern changes prior to particle bouncing, and QL approximation remains valid. Conversely, the particle is *trapped* and the linearization fails for too high RF power density, *p*_RF_.

**Figure 5:**
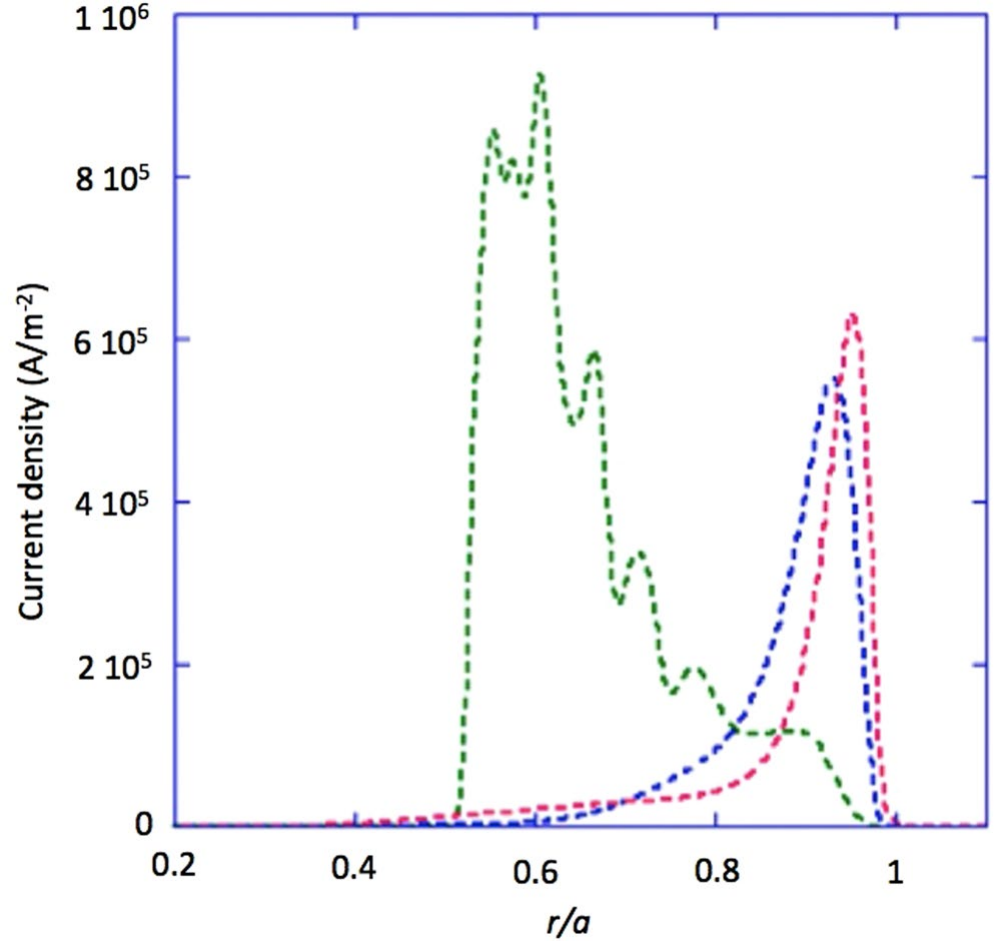
Radial *j*_LH_ profiles modelled by inputting kinetic profiles of Fig. 3 relevant to the pulsed reactor regime, and antenna spectra of Fig. 4 with: Δ*n*_//_ = 0.085 (green curve), Δ*n*_//_ = 0.33 (blue curve) and Δ*n*_//_ = 0.58 (red curve). The effect of the minor lobes present at negative and positive *n*_//_ of the launched antenna spectrum are included.

Consequence of the numerical results of the LH-driven current density profiles (shown more ahead referring to the *j*_LH_ profile shown in Fig. 5, green curve, regarding case of narrowest spectrum of Fig. 4), the phase velocity width is (∼0.17*c*) found much larger than the trapping velocity width (∼0.002*c*), for *p*_RF_ ≈ 30 MW/m^2^. Realistic values of the minimum and maximum phase velocities of the propagating wave spectrum, consistent with production of EDF plateau, have been calculated near the peak of absorption radial layer (*r/a* ≈ 0.6, as obtained by ray-tracing and Fokker Planck analyses reported more ahead in regard to the modelled *j*_LH_ profile shown in Fig. 5, green curve). A wave electric field with intensity (0.2 kV/cm) markedly larger than that (of 0.05 kV/cm) expected to occur at that layer, has been considered, which overestimates the trapping velocity width and, consequently, proves validity of QL theory with larger margin.

### Parasitic phenomena of spectral broadening

It is hereafter assessed the role of parasitic phenomena of parametric instabilities (PI)^13–15,29^ and density fluctuation linear scattering (DFLS)^30–32^ of LH waves.

These mechanisms can broaden the width of the launched antenna spectrum and would frustrate the intent of enabling LHCD in hot plasmas. Details for modelling these undesired effects, which should be however accounted for modelling the *j*_LH_ profiles in reactor plasmas, are summarised in the Method Section.

PIs in magnetized plasma are non-linear phenomena occurring when the coupled RF power (*pump wave*) exceeds a certain threshold in *p*_RF_. Low frequency evanescent modes of the thermal background of density fluctuations drive this mechanism, establishing an energy and momentum transfer from high to low frequencies of plasma modes. This leads further LH waves (sidebands) to growth from the noise level, which frequencies and refractive indices shifted by certain amounts from the nominal *ω*_0_ and *n*_//0_ of the launched spectrum.

Since the spectral broadening effect is generally larger in colder plasmas of the radial periphery of the column^13–15^, different *T*_e_ profiles of edge plasma have been considered for assessing conditions that make negligible the PI effect. This occurs in case cold plasma (of several eV) is limited within a small antenna–plasma distance (≦3 cm), which is reasonable assumption for hot reactor plasmas. The residual PI contribution of spectral broadening has been however retained in modelling the *j*_LH_ profiles. In this regard an exhaustive work devoted to assess the impact of PI-produced spectral broadening in reactor relevant conditions has shown that negligible effect is expected to occur, and successful LHCD in reactor plasmas is consequently enabled^33^.

The DFLS mechanism can alter the launched spectrum via random rotation of the refractive index in a plane perpendicular to the magnetic field. The local dispersion relation, $\omega =f({n}_{{|}{|}},{n}_{{\perp }},r)$, requires that both the components of the refractive index should be conserved.

The DFLS contribution of broadening of the launched antenna spectrum is described by^30^:

5 $${k}_{//}\approx \frac{Aq}{{k}_{\perp }x+{k}_{//in}}$$

In Eq. 5, *A* is the aspect ratio defined as: *R*_0_/*a*, *q* is the safety factor (see the Introduction), and *k*_//in_ represents the wavevector component value before initiating the scattering process. The amplitude of the density fluctuations can be estimated from the mixing length criterion as $\delta {n}_{e}/{n}_{e}\cong {(\xi {L}_{n})}^{-1}$ where ${L}_{n}$ is the density gradient scale length^18^.

Considering DEMO parameters, the spectral broadening effect on the narrower spectrum of Fig. 4 (Δ*n*_//_ = 0.085) has been evaluated. Consequently the DFLS effect is very different in the cases of coupling the LH power from the low or high field sides: in the former, Δ*n*_//_ increases strongly (from 0.083 to about 0.20), mostly at distances of several centimetres from the antenna mouth, whilst the effect is negligible for high-field side launch.

Therefore, depending on the *q*-profile, the change of the poloidal wave number due to the rotation of the perpendicular wave number affects the ray-tracing pattern and the *n*_//_ power spectrum. For density perturbations with poloidal wavenumber of the order of 0.1ρ_i_ (thermal ion Larmor radius), the analysis results suggest that the DFLS spectral broadening effect is negligible for *r/a* > 0.85 but can be sufficient to prevent the LH power penetration in the plasma core (*r/a* < 0.6).

For density fluctuations with amplitude a factor four larger than expected by the mixing length criterion, the effect of spectral broadening should not be longer neglected. However for such large level of turbulence it might be problematic also to achieve the goal of a sufficiently low transport in a reactor.

### Modelling of the current profile

The LHCD modelling have been performed considering a RF power *P*_RF_ = 80 MW, corresponding to *p*_RF_ ≈ 30 MW/m^2^ launched from the high-field side of the DEMO plasma. The results are shown hereafter.

Figure 5 shows the *j*_LH_ profiles obtained considering the *pulsed* reactor regime of Fig. 3. For narrower spectrum (Δ*n*_//_ ≡ 0.083), the profile extends to the inner radial half of plasma (*r/a* ≳ 0.4, where the current density is: *j*_LH_ ≈ 1.2MA/m^2^). The current density gradually displaces more off-axis for broader spectra (at *r/a* ≳ 0.6 for Δ*n*_//_ ≡ 0.33, and at *r/a* ≳ 0.8 for Δ*n*_//_ ≡ 0.58). For the case obtained considering the *pulsed* case of Fig. 3, and narrower spectrum (Δ*n*_//_ ≡ 0.083), a slightly inner deposition is obtained with respect to case of *steady-state* regime of the figure. This is due to the higher *T*_e_ values, see Fig. 3b, occurring at around mid radius (*T*_e_ ∼ 16 keV in front of *T*_e_ ∼ 14 keV for the pulsed case), which facilitates the wave damping accordingly with Eq. 1.

The total driven current is of about 4.4 MA. In both cases, the LHCD efficiency^9^, defined as: $$\eta ={R}_{0}\langle {n}_{e}\rangle $$$$\frac{{I}_{CD}}{{P}_{abs}}(\frac{Ampere}{Watt\times {m}^{2}})$$, is: *η*_LHCD_ ≈ 0.3AW^−1^m^−2^.

By including in the narrowest antenna spectrum of Fig. 4 the strongest broadening effect produced by PI, only minor changes of *j*_LH_ profiles, driven current and absorbed power have been obtained.

In order to evaluate the effective LH-driven current, the antenna directivity parameter should be considered, defined as: $dir=\frac{{P}_{+}}{{P}_{+}+{P}_{-}}$, where *P*_+_ and *P*_−_ indicate, respectively, the wave power fraction travelling in the co- and counter plasma current direction. Consequently, *dir* ≈ 60% has been found.

Further numeric run has shown that using a even narrower spectrum (Δ*n*_//_ ≡ 0.070, still compatible with QL theory limit, and enabled by antenna hardware (see the Method Section), has the effect of greatly reducing current drive, which makes this choice counterproductive. Such effect is explained in the following way. For sufficiently large power density, as occurs for narrow spectra with Δ*n*_//_ < 0.1 considered here, the current density driven is roughly proportional to the width of the *n*_//_ power spectrum, and the dependence of the quasilinear diffusion coefficient on the power density becomes marginal. In this limit, indeed, the distribution function vs. *u*_//_ can be assumed flat in an interval of velocities proportional to Δ*n*_//_. For larger spectral width, Δ*n*_//_ ≳ 0.2 the quasilinear diffusion coefficient becomes smaller thus compensating the effect of the spectral width^9^.

**Figure 6:**
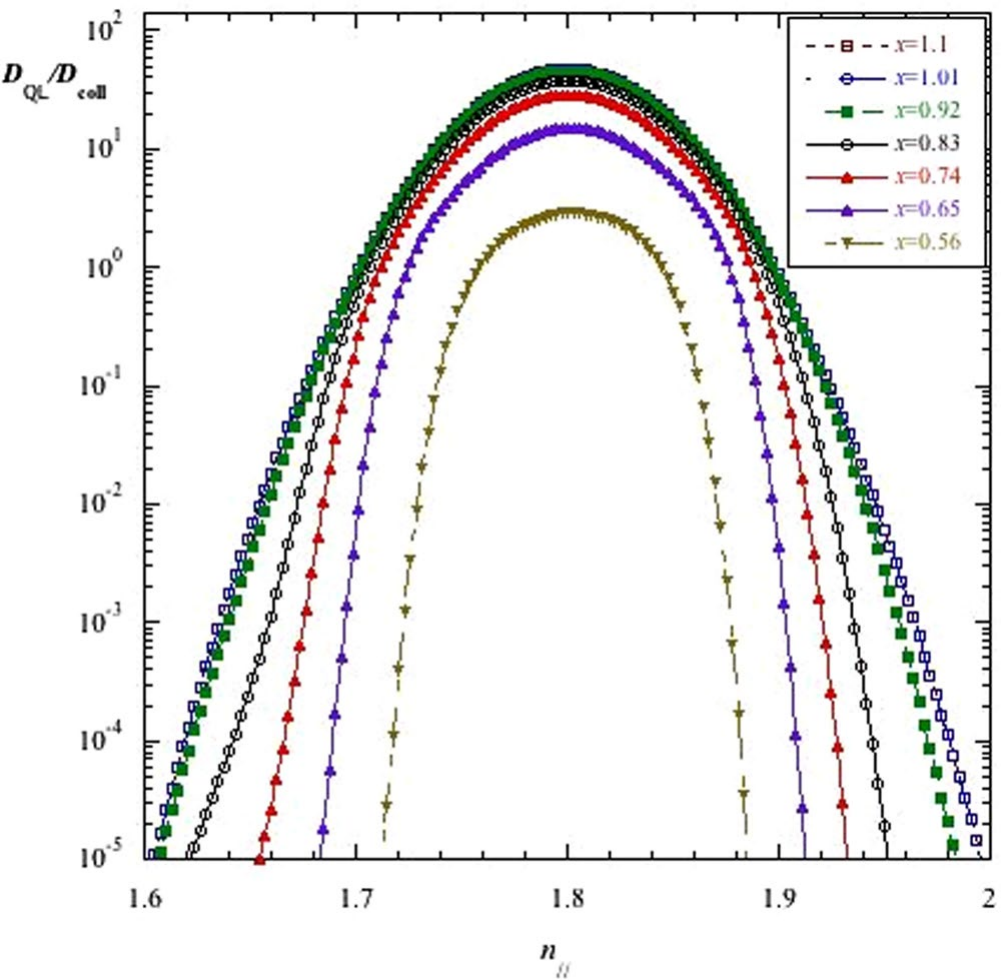
Evolution of the quasilinear diffusion coefficient normalized to the collisional diffusion plotted versus the parallel refractive index at the antenna and for various radial location quasi-linear diffusion coefficient plotted versus *n*_//_, for different radial positions. Note that lines regarding the radial positions *x* = 1.10 and *x* = 1.01 are quite overlapped and cannot be distinguished. Antenna spectrum: Δ*n*_//_ ≡ 0.083. Other parameters as in Fig. 5.

The series of well-distinguished peaks in the *j*_LH_ profiles of Fig. 5 in the cases of narrow antenna spectrum (Δ*n*_//_ ≡ 0.083) cannot be attributed to numerical instabilities. Results have been verified, indeed, to be robust however changing the size of the numerical mesh. Moreover the radial evolution of the quasi-linear diffusion coefficient for the same case of narrow spectrum exhibits a regular trend at any radial positions as required by quasilinear theory, shown in Fig. 6.

Considering profiles for *steady-state* regime of Fig. 3, by narrow spectrum (Δ*n*_//_ ≡ 0.083) and lower *n*_//0_ value (≈1.7 in place of 1.8, which is possible thanks to larger margin of the LH wave accessibility condition^11^), slightly radially inner LHCD is produced, with also higher *j*_LH_ value (of about 20%). The main *j*_LH_ peak usefully displaces at *r/a* ≈ 0.54 from *r/a* ≈ 0.60 for case of Fig. 5.

We have considered the case of ITER displayed in Fig. 3, but with spectra of Fig. 4 centred at *n*_//0_ = 2 and *P*_RF_ = 24 MW, and same antenna design of ref.^28^. Importantly, keeping fixed *n*_//0_ and setting the antenna spectrum with Δ*n*_//_ in a suitable range (from Δ*n*_//_ = 0.083 to Δ*n*_//_ = 0.50, as enabled by hardware), the *j*_LH_ peak can usefully span in the outer radial half of plasma (respectively, from *r/a* ≈ 0.55, where *T*_e_ ≈ 14 keV, to *r/a* ≈ 0.64, where *T*_e_ ≈ 8 keV). This corresponds to markedly increase (up to ∼15%) the *j*_LH_ tailoring flexibility obtained by previous know how^34^ (<4%). Acting also on the *n*_0//_ parameter, flexibility is further increased (the layer *r/a* ≈ 0.80, where *T*_e_ ≈ 3 keV, is reached using *n*_0//_ = 3.2, in place of *n*_//0_ = 2, and Δ*n*_//_ ≈ 0.15). The driven total current is of about 1MA.

By including the DFLS effect assessed for the case of ITER, owing to the relatively low value of the toroidal magnetic field in the low-field side location of antenna, the spectrum results markedly broadened (from Δ*n*_//_ = 0.083 to Δ*n*_//_ = 0.60, for the narrower nominal case). This problem further imposes that both the Δ*n*_//_ and *n*_//0_ keys should be exploited for compensating the undesired DFLS effect of spectral broadening and enabling an efficient and flexible current profile shaping by LHCD in ITER, otherwise impossible by previous understanding^34^.

A wide scan of input parameters, in terms of different plasma density and temperature profiles, and antenna spectra, also modified by plasma edge physics effect, has been performed with the aim of assessing robustness of the obtained results^33^. These results indicate that the modelled *j*_LH_ profile would be tailored by acting on the Δ*n*_//_ antenna parameter.

About uncertainties of analysis, the *j*_LH_ profile depends mostly on the *T*_e_ profile. Consequently, change of 10% of *T*_e_ profile modifies of same amount the *j*_LH_ profile. Conversely, much bigger changes of other input parameters (up to 40%) determine only minor changes of the *j*_LH_ profile (however satisfying the LH wave accessibility condition^11^).

Further numerical results shown hereafter allow assessing the role of the RF power density, *p*_RF_, and Δ*n*_//_ antenna parameter in determining the RF power absorption.

### Antenna parameters and LHCD profiles

It is useful to compare modelling outcomes obtained considering the same input parameters referring to results displayed in Fig. 5, but in the simpler framework of the linear wave theory^11^ instead of the QL limit^18^. This way, the complex interaction occurring between the wave spectral components and the EDF is voluntarily neglected. The comparison allows evidencing that quasilinear effect plays a key role for tailoring the current profile.

Focussing on changes occurring for the narrow spectrum case of Fig. 5 (Δ*n*_//_ ≡ 0.083, green curve), in the linear theory limit, the main *j*_LH_ peak is found to be dislocated markedly more externally (at *r/a* ∼ 0.9) than in the QL framework (*r/a* ∼ 0.5) taken as reference.

In the QL limit we have identified what antenna parameter, among *p*_RF_ and Δ*n*_//_, is able producing more significant changes in the current density. For this aim, the *j*_LH_ profile for narrower spectrum of Fig. 5 has been recalculated considering a lower value of *p*_RF_ (≈3 MW/m^2^ in place of 30 MW/m^2^). Consequently, the main peak of *j*_LH_ dislocates only slightly more externally (from *r/a* ∼ 0.5 to *r/a* ∼ 0.6). Further larger values of *p*_RF_ (>30 MW/m^2^), which however should not be recommended for safe antenna operations, reflect in minor changes of the current profile.

Therefore, the QL effect of the spectral width shown in Fig. 5 plays safer and more useful role than larger *p*_RF_ in reducing the wave damping and, consequently, favouring more intensely the penetration of the wave packet into hot plasmas, as necessary in a reactor.

## Discussion

The method presented here provides larger flexibility in tailoring the current density profile in reactor plasmas by the LHCD method. For this purpose it is necessary the exploitation of three key antenna parameters, Δ*n*_//_, *n*_0//_, and *p*_RF_, in place of only the two latter as only possible by previous know how^17,34^. Using both the Δ*n*_//_ and *n*_//0_ parameters allows to flexibly shaping LHCD profiles also in the hottest reactor plasmas envisaged so far. The efficiency is also larger than with other CD tools^1,3,16^.

In order to make negligible the broadening of the launched antenna spectrum via DFLS effect, analytical results support the conclusion that the antenna mouth should be located in a plasma region of DEMO where a relatively high value (>8 T) of the confinement magnetic field occurs, i.e., in the high-field side. Operations proposed with lower *B*_T0_ (≲6.5 T)^35^ are consequently not recommended.

For ITER, lower electron temperatures are envisaged than in DEMO (see Fig. 3), making less problematic the LH power penetration into the main plasma region. However, in order to face undesired DFLS effect of spectral broadening, the exploitation of the new key antenna parameter, Δ*n*_//_, should be mandatory. General conclusions on LHCD exploitation in the plasma reactor can be drawn because of the greater dependence of jLH profile from the electron temperature profile.

On the light of the present breakthrough, the high value of *B*_T_ (>9 T) proposed for ARC^36^ is appropriate for providing full flexibility of the LHCD tool. Higher magnetic field is useful for reducing not only the DFLS effect resulting by wave propagation, but also the intrinsic amplitude of fluctuations. Indeed the latter is roughly estimated by the mixing length criterion as: $\delta {n}_{e}/{n}_{e}\cong l/{L}_{n}$, where *L*_*n*_ is the density gradient scale length and *l* is the perpendicular scattering length of fluid or plasma element due to the fluctuations^18^. Their level is thus proportional to the perpendicular coherence length of the fluctuations, which is in turn proportional to the ion Larmor radius. Therefore, smaller amplitude plasma fluctuations are expected on the high field side of tokamak plasma.

Results obtained with the LHPI code^15^ for the hypothesised DEMO plasma show that parametric instabilities produce negligible parasitic effect of spectral broadening. The latter was avoided, indeed, tough in FTU experiments with relatively cold plasma at reactor relevant densities^12^. Thus, a fortiori, high *T*_e_ envisaged at large radii of reactor plasmas would naturally depress PI effects and enable LHCD.

As further undesirable cause of broadening of the launched *n*_//_ antenna spectrum, diffraction effects might be originated by non-uniform distribution of the coupled radiation in the poloidal direction and by presence of caustic in the poloidal plane^37,38^. This can broaden the power flux spectrum coupled to a certain poloidal wavenumber and, consequently, also the *n*_//_ spectrum would broaden in proportion to the ratio of the poloidal and the toroidal magnetic field. The effect entity depends on the detail of the poloidal distribution of the LH radiation beam and can be determined by a full wave analysis, which is however problematic for huge cpu-calculation time imposed by the sizes of reactor geometry. It has been estimated a upper limit of diffractive spectral broadening based on the poloidal focalisation determined by ray-tracing^39^. Consequently the poloidal angle extension of the plasma region illuminated by a single toroidal array of waveguides is reduced to not less than 70% of the initial one when the beam reaches the most internal area corresponding to *r/a* ≈ 0.4. Considering a reciprocal broadening of the spectrum in a poloidal wavenumber equal to a factor of 1.4, and because the ratio between the poloidal and toroidal component of the magnetic confinement field is certainly less than 10%, a maximum limit of 15% of *n*_//_ spectral broadening has been found. We are thus confident that between the first surface (where the wave-spectrum is established inside the plasma as indicated by the used coupling code^39^) and the absorption layer, diffraction plays a negligible role.

The following information further supports result robustness of the numerical tool utilised here. The same tool was used also to obtain successful predictions and interpretations of data of milestone experiments of JET^40^. They demonstrated the fundamental LHCD capability of sustaining a phase of high thermal insulation of plasma, in reactor-relevant condition of high *T*_e_ of plasma core producing full deposition of the LHCD power at first half of radial pass^13,14^. Agreement with current profile evolution from diagnostic measurement was found thanks to physics of plasma edge that the LH^star^ suite of codes includes with respect to other approaches (see the Method Section). The importance for *j*_LH_ modelling of retaining the effect of spectral broadening as done previously^13^ has been recently acknowledged^41^.

In further experiments of JET with relatively high plasma density at large radii, the use of the LH^star^ tool allowed interpreting the observed lack of penetration of the coupled LH power into the main plasma as effect of too marked PI-produced broadening of the launched *n*_//_ spectrum^42^. Successful interpretation of similar phenomenology observed on FTU was also provided^12,15^.

The LH^star^ package of numerical codes has the special feature of respecting the geometric optic (WKB) limit at the basis of the model. For this purpose only the reactor relevant singe-radial pass regime of LH wave propagation is considered for robust modelling. In case of multi-pass regime the LH^star^ tool formulates a guess about the toroidicity effect in modifying the propagating *n*_//_ spectrum^12,15^, and the modelling cannot be considered robust. Indeed, in the multi-radial pass regime the WKB limit fails owing to occurrence of LH wave cut-off located at the plasma edge. From these layers the supposed rays would be reflected back although the location of such reflection is undetermined indeed. Other approaches generally used for LHCD modelling^43–45^ derogate from this important constraint and seek to however consider the multi-radial pass regime, which often occurs in tokamak experiments. This is done by means of complicated architectures that, besides being recognised to violate the WKB limit^41^ might make difficult to display the role of the Δ*n*_//_ antenna parameter in tailoring the *j*_LH_ profile, as instead done here. Furthermore, the same LH^star^ package enabled performing predictions^13,14^ later confirmed in FTU experiment, which has successfully extrapolated LHCD to reactor-relevant high plasma densities^12,15^.

The operating frequency of 5 GHz considered here is high enough to minimize the possible damping on fusion born alpha particles^44–46^, and is in the range of the available high power sources^47,48^. Lower operating frequencies would enhance the alpha particle effect^45,46^. Although using a higher LH operating frequency may be technically problematic, numerical results obtained for the same cases of reactor relevant plasmas presented here, but considering a higher frequency (of 8 GHz in place of 5 GHz), indicate only a marginal improvement of the RF power penetration thanks to larger margin of LH wave accessibility condition^11^. Consequently the benefit of reducing the spectral width should be much largely more preferable.

The antenna modules (whose geometry is described in the Method section) is derived from the well-known Bibet’s project of LHCD antenna for ITER^28^, and the necessary electronics can be made with bearable efforts. The Δ*n*_//_ and *n*_//0_ key antenna parameters can be electronically set with good precision (∼1%) and stability, and speed is much faster than the characteristic time of relevant plasma instabilities to keep under control. Implementation of the LHCD tool in a current profile control system, which a reactor mandatorily requires, is thus now possible.

The breakthrough presented here proves that the LH power deposition should not be, indeed, too far out in the DEMO plasma, as previously found^16,17^. For a reactor, a current profile control system should be now envisaged, whose coverage would include the whole outer radial outer half of plasma, in particular, the pedestal radial layer that is crucial for stability [M. Shimada, *et al.*, Chapter 1: Overview and summary, Editors of ‘Progress in the ITER Physics Basis’: Nucl. Fusion 47 (2007) S1–S17]. Self-consistent modelling including transport effect should be useful in a second step of analysis. However, in regard to LHCD profiles of Fig. 5, if imagined initiating an evolving regime of reactor plasma, the current profile control system should precisely accomplish the task of tailoring the CD profile to face the consequences of unpredictable changes of the kinetic ambient profiles, their impact on the self-produced current density profile and, in turn, on confinement and stability. The LHCD option should be thus reasonably reconsidered as a priority for ITER and DEMO, in order to help facing the challenge of a fusion gain developing in a context free from plasma instabilities.

## Conclusions

A reactor needs undoubtedly of actively shaping the current profile at large radii of the plasma column for exploiting the self-produced current and saving the huge costs of the heating and CD systems. In addition, a fascinating complexity of intercommunicating mechanisms should be established, leading to burning plasma via hopeful govern of still unknown instabilities^7^.

The natural current profiles produced by the plasma are usefully matched by lower hybrid current drive, successfully extrapolated to high densities and, now, also with larger flexibility, to high temperatures envisaged for a thermonuclear reactor, thus enabling his economic viability.

## Methods

We show the main parameters considered for the LHCD analysis for DEMO, and details of the approach followed for assessing the configuration of the antenna at the plasma interface, and the numerical tool utilised for modelling the LH-driven current density profile.

### Main plasma parameters

The assumed DEMO plasma^16,26^ has toroidal shape with: major radius on the axis of the column: *R*_0_ = 9 m, minor radius on the equatorial plane: *a* = 2.25 m, toroidal magnetic field: *B*_T0_ = 6.8 T on the axis. In the *pulsed* regime case, the plasma current is: *I*_P_ = 18 MA. In the *steady-state* case is: *I*_P_ = 22 MA.

### Antenna configuration

The antenna has been hypothesised on the basis of the same modules of the LHCD project proposed for ITER^28^, consisting in modules of rows combined along the toroidal and poloidal direction. A single row (aligned to the toroidal direction) is formed by 24 couples of active-passive waveguides, and an additional passive waveguide (i.e., not fed) is placed at the beginning of the row in order to have passive waveguides at the ends. Therefore, 24 active waveguides and 25 passive waveguides in this row have been assumed.

The antenna spectra have been numerically calculated by using the GRILL-3D module^26^ of the LH^star^ package described more ahead. The peak value *n*_0//_ = 1.8 of the spectra in Fig. 3 has been obtained considering a single row using waveguide width of 9.5 mm, height 58 mm, wall thickness 3 mm, and phase shift between active waveguides of 90 degrees. The size of the row is of 615.5 mm.

The *n*_//_ peak value can be changed using different waveguide width that, increased at 12 mm, would produce a spectrum with lower peak, *n*_0//_ · 1.5 and, reduced at 8.25 mm, produces a spectrum with a higher peak, *n*_0//_ · 2.0. Furthermore, with the waveguide width of 9.5 mm, the *n*_0//_ value should be increased by using lower phasing value for active waveguides. For instance, *n*_0//_ increases to 1.96 for phasing of 65 degrees, and to 2.1 for 45 degrees. It is necessary to design a suitable antenna structure that allows this phasing.

The full antenna size in the toroidal direction is 1215.5 mm. By feeding together identical modules piled along the poloidal direction, the spectrum does not change but the coupled power increases. A power of 80 MW can be coupled by an antenna consisting in a module pile of 37 rows, at a frequency of 5 GHz allowed by available RF power sources^45,46^. The power density is (30 MW/m^2^) far from breakdown limit (70 MW/m^2^ at 5 GHz) found in LH experiments in tokamaks^24^.

The spectral width should be minimized (at Δ*n*_//_ ≈ 0.07) by setting the phase of 90 degrees between adjacent active waveguides. The two spectra of Fig. 3 with small and large width (Δ*n*_//_ = 0.083 and Δ*n*_//_ = 0.58) have been obtained by changing the number of fed modules.

The power reflected back to RF generator can be kept low under a quite large variation of the plasma edge density occurring during operations, by using a passive-active multi-junction (PAM) antenna with one or more bi-junction planes^28^.

### Modelling tool for the lower hybrid current drive profiles

The LH-driven current density radial profiles are modelled utilising the LH^star^ package of numerical codes, consisting of three computation sub-tools: *i*) the code GRILL-3D^27^, capable of calculating the spectra and any relevant parameter of waveguide antennas, with also very complicated geometries and for any feeding and phase conditions; *ii*) the LHPI (lower hybrid parametric instability) code^15^, based on first principles of non-linear physics of parametric instabilities occurring at the plasma edge, which calculates the PI-produced spectral broadening of the launched antenna spectrum^13^, *iii*) the RAY^star^ module^49^ that utilizes the spectrum initially broadened by combination of PI and DFLS effects, and performs the ray-tracing in toroidal geometry. The RAY^star^ code calculates, at any radial layer interested by RF propagation in the plasma, and for any *n*_//_ spectral component, the effects of wave propagation in toroidal geometry that can further broaden and upshift the LH wave spectrum^22^. Therefore, at each radial step, the quasi-linear diffusion coefficient is assessed considering the 2-D Fokker-Planck relativistic equation solved for the electron distribution function in velocity space, and the quasilinear damping is consistently taken into account in the LH wave power equation. On this basis, the RF power density and LH-wave-driven current density radial profiles are calculated.

Ray-tracing analysis consists in solving the equation system for the position and wave vector allowing the reconstruction of the wave-phase and the RF power damping rate along the trajectory^11^. Since the RAY^star^ code utilises ray-tracing approach, the geometric optic limit (WKB) approximation must be respected. Consequently the propagation region must exclude the LH wave cut-off layers located at the plasma edge^11^. This constraint imposes that cases of weak damping per single pass, producing multi-radial pass regime that occurs in most tokamak experiments performed so far, cannot be considered, and a guess of the toroidicity effect up-shifting the propagating *n*_//_ antenna spectrum should be guessed, as originally done for interpreting outcomes of LHCD experiments^9,49^.
